# A prospective clinical study on implant impression accuracy

**DOI:** 10.1186/s40729-019-0190-6

**Published:** 2019-11-19

**Authors:** Motaz Osman, Hassan Ziada, Ahmed Suliman, Neamat Hassan Abubakr

**Affiliations:** 1 Department of Oral Rehabilitation, Faculty of Dentistry, University of Khartoum, Khartoum, Sudan; 2 Clinical Sciences, School of Dental Medicine, University of Nevada, Las Vegas, NV USA; 3 Department of Oral Maxillofacial Surgery, University of Khartoum, Khartoum, Sudan; 4 Biomedical Sciences, School of Dental Medicine, University of Nevada, Las Vegas, 1001 Shadow Lane, Suite 240, MS 7412, Las Vegas, NV 89106 USA

**Keywords:** Implant impression accuracy, Open tray technique, Closed tray technique, Marginal discrepancy

## Abstract

**Background:**

An accurate impression is crucial to the long-term success of dental implants. This investigation evaluated the accuracy of the open and closed implant impression techniques in partially edentulous patients who received two adjacent implants.

**Material and methods:**

Forty patients received Osstem Implants (Osstem Implant System, Seoul, Korea). Two impressions were made for each patient, one using an open tray and a second with a closed tray technique. The horizontal distances between two impression copings were measured and compared to similar measurements on the master casts. Also, under a stereomicroscope (AmScop14370, Myford Road, #150, Irvine, CA 92606 USA) at a 50-fold magnification, the presence or absence of the marginal discrepancies was evaluated.

**Results:**

There were no statistically significant differences regarding horizontal measurements and in the marginal relationship for the two impression techniques, except between the anterior and posterior regions, for the closed tray technique. There were also no statistically significant differences in the impression accuracy between maxillary and the mandibular arches. In addition, there were no statistically significant differences for the intraoral horizontal distances, compared to similar horizontal measurements on master casts, between the open and closed tray techniques.

**Conclusions:**

Within the limitations of the present study, there were generally no differences in the impression accuracy between the open and closed tray techniques in partially edentulous patients with two adjacent implants.

## Introduction

Tooth loss reduces the masticatory ability, compromises esthetics, and may consequently diminish social interactions, which could significantly impact on the quality of life of individuals [1–3]. Treatment options for teeth loss are continuously evolving, from the removable prosthesis to the increasing preference for fixed choices. Furthermore, the progress in the manufacturing of titanium implants added to their long-term success, increasing the fixed prosthetic options for replacement of missing teeth, making implants an essential part of contemporary dental practice and a popular choice for both patients and clinicians [4].

An implant impression is primarily a three-dimensional record of the implant and the surrounding tissues. Impression accuracy is a significant factor in implant long-term success. Inaccuracies or errors occurring at any stage of the superstructure construction may lead to a lack of precision fit between various components. The lack of potential compensatory readjustment, due to the absence of intervening periodontal ligament, may have the consequence of related complications or failure [5, 6].

The fit of an implant superstructure is considered “passive” if it does not create or lead to any static loading within the prosthesis, or in the surrounding bone. Imperfections in the precision fit may increase the incidence of mechanical problems or abutment loosening as well as possible fracture of the prosthetic or implant components. Furthermore, any resultant marginal discrepancies as a consequence of inaccurate impressions may enhance plaque accumulation, which would impact negatively on the soft and hard tissues around the implant [7].

The research on implant impression accuracy is mostly from in vitro studies, and the limited number of clinical studies might be contributing to the controversy as to which technique should be considered to be more superior [5, 8]. We hypothesize that clinically, there is no impact or differences in impression accuracy.

This study aimed to evaluate the accuracy of the open and closed implant impression techniques in partially edentulous patients with two adjacent implants.

## Materials and methods

The current study was conducted to investigate the accuracy of the open and closed implant impression techniques in partially edentulous patients with two adjacent implants. The ethical principles were adhered to, and ethical approval to conduct the study was duly obtained from the Ministry of Health, State Khartoum, Khartoum University Teaching Hospital, number: [WK/OS/AETEA/44/1].

Patients who were scheduled to receive two adjacent implants were invited to participate in the study. The sample that would have sufficient power for analysis was calculated based on data from the previous clinical study by Stimmelmayr in 2013 [9]. The sample size was determined using the following formula:

$$ n=\frac{{\left(\  z\sigma \right)}^2}{(d)^2} $$

where:

*n* = the required sample size

*Z* = is the critical value of the normal distribution

*σ* = the standard deviation taken from the previous study

*d* = the margin of error (10% × mean).

The sample size was 31 patients; this was increased to 40 participants to accommodate patient dropouts during the study.

The inclusion criteria were patients over 18 years of age and willing to participate. A prerequisite to participation was a treatment plan that would involve two adjacent implants. The patient should also be category ASA I or ASA II medical history (American Society of Anesthesiologists Classification) [10]. Furthermore, evidence of bone loss or implant mobility at the time of impression making, formed part of the exclusion criteria [11, 12].

**Fig. 1:**
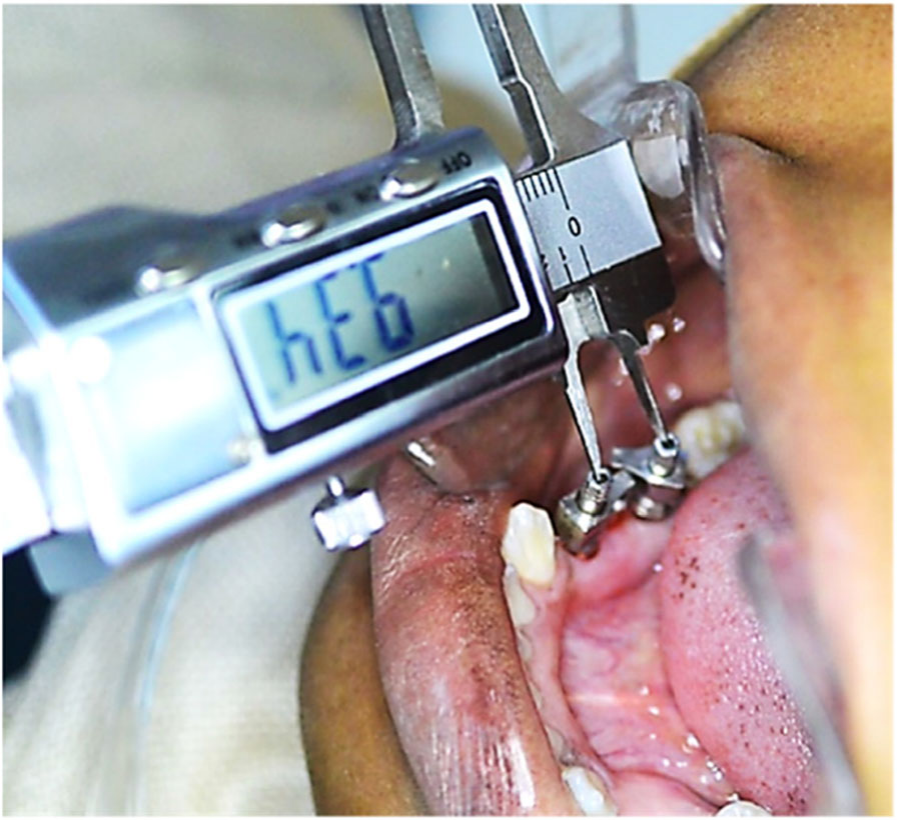
Horizontal measurements between the two impression copings in the patient’s mouth

Informed consent was made, and participants who agreed to participate signed the consent form. For every patient, a surgical positioning guide was fabricated from a diagnostic wax-up that correlated the anatomic conditions. The implant (Osstem Implant System, Seoul, Korea) installation directions were carried out according to the amount and status of the available bone [13]. A Specialist Oral Surgeon placed the implants using the manufacturer’s standardized technique, and similarly, a Specialist Prosthodontist carried out the related restoration steps. For making the impressions, individual trays were initially checked intraorally, and the final impressions made using Virtual Monophase vinyl polysiloxane impression material (Ivoclar Vivadent AG). Before impression making, the horizontal distance between the two impression copings was measured inside the patient’s mouth using a digital caliper (HSL 246-15, Karl Hammacher GmbH, Germany) and recorded (Fig. 1). This recorded intraoral horizontal distances would later be compared against similar horizontal measurements on the master casts, to evaluate discrepancies or horizontal displacements between the positions intraorally and on the master casts. The same impression evaluation criteria used in our previous study were also used here [8], which was described by Lee and Gallucci as follows [14]:

1. There should be accurate imprints of the implant areas.
2. There should be no voids in the occlusal, buccal, lingual, and interproximal surfaces of the neighboring teeth.
3. There should be a proper reproduction of the implant area.
4. There should be no impression material in the analog-impression coping interfaces.
5. The impression material should not be separated from the custom tray.
6. The transfer copings should not be displaced from the impression.

Any impression not meeting these criteria was repeated until the criteria were met. Two impressions were made for every patient by the same clinician, one using the open and a second with the closed tray technique.

**Fig. 2:**
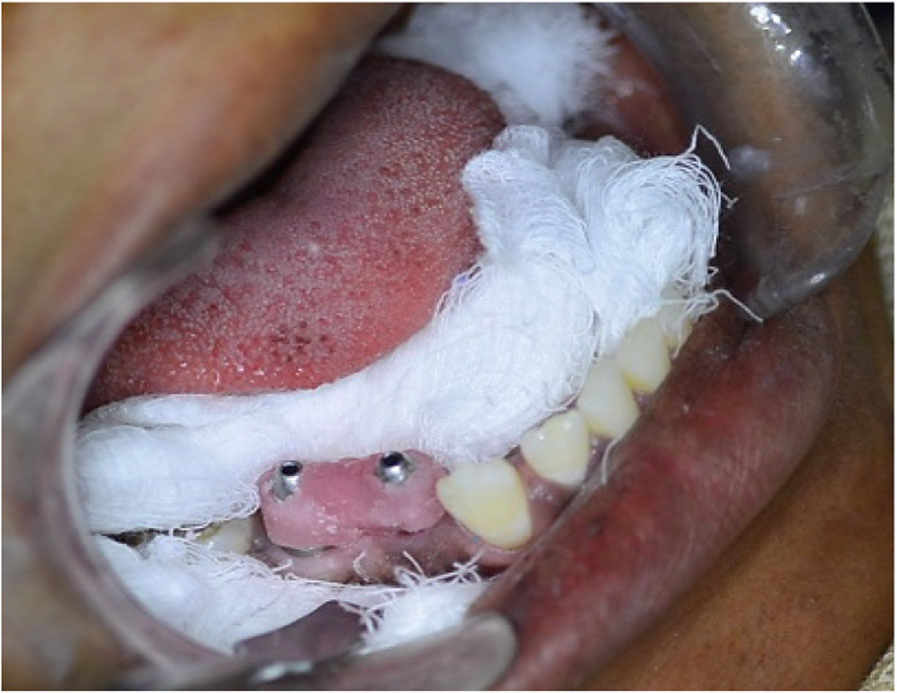
Light cure acrylic resin verification jig in the patient’s mouth

For the analysis of accuracy in the vertical direction or marginal discrepancy, verification jigs were constructed to connect the two impression copings [15]. These verification jigs were used to transfer the relationships between the two impression copings and their implants from the patients’ mouths to the master casts. To construct the verification jig, the two impression copings would be attached to their implant, inside the patient mouth, and a string of dental floss is wrapped around to connect the two impression copings (Fig. 2). A light cure acrylic resin (Al dente dental products GmbH, Germany) adapted over the dental floss in increments and cured according to the manufacturer’s instructions. The impression coping’s screws would then be loosened, and the jig removed [16].

The impression copings for both the open and closed tray techniques were re-assembled and fixed into their corresponding implant analogs. Dental Stone Type IV (Elite Rock, Zhermack) was mixed using a vacuum machine for 30 s, then poured using the boxing technique over a vibrator, and the casts separated after 45 min according to the manufacturer’s instruction [17, 18]. The master casts were then sectioned to a base of 20 mm, to allow their allocation under the stereomicroscope (AmScop14370, Myford Road, #150, Irvine, CA 92606 USA), to be examined at a × 50 magnification, and to evaluate the presence or absence of marginal discrepancy [8, 19].

Two examiners were involved in the evaluations, and inter-examiner reliability of 0.932 was obtained.

### Statistical analysis

All the data were tabulated and statistically analyzed using IBM SPSS Statistics software version 22. The *t* test was used to compare intraoral and master cast horizontal distances as well as sub-groups of the open and close impression tray techniques. Where data are not normally distributed, Wilcoxon signed test was used for numerical dependent data and paired data; Mann-Whitney *U* test was used for independent numerical groups. Chi-square test was used for the association between categorical variables. The *p* value was set at *p* ≤ 0.05 and regarded as statistically significant.

## Results

**Fig. 3:**
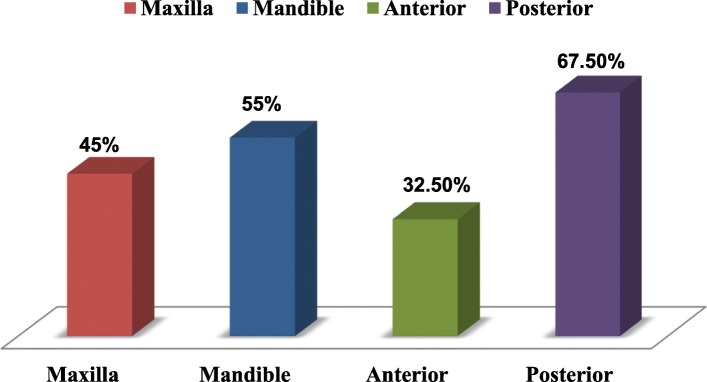
Sample distribution according to arch and position

Eighty impressions were made for 40 patients, using the open, then the closed impression techniques. There were 18 impressions in the maxillary and 22 in the mandibular arch; of these, 13 were in the anterior and 27 in the posterior region (Fig. 3).

**Fig. 4:**
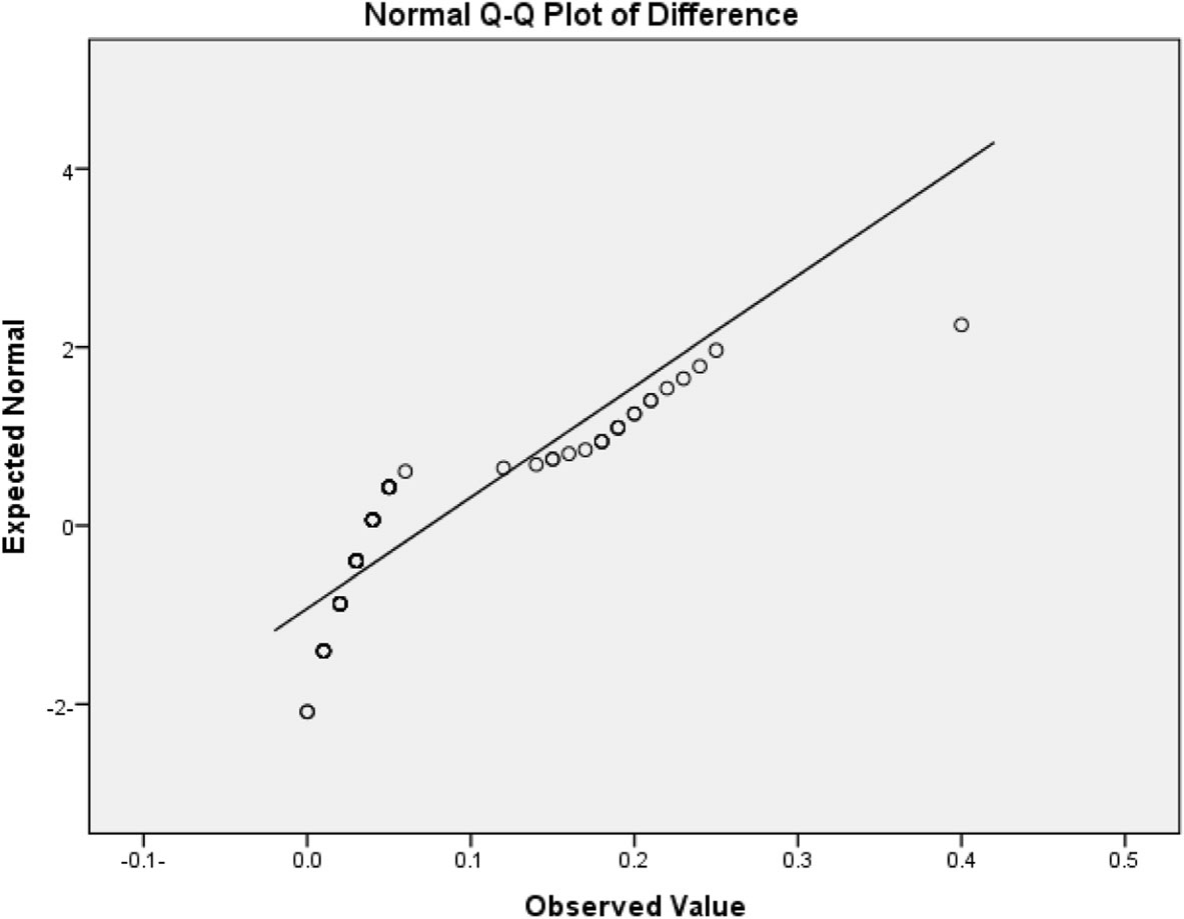
Normality line of the distribution horizontal measurement data for the intraoral and working casts

A *t* test indicated no statistically significant difference between the open and closed tray techniques for intraoral horizontal measurements against the similar horizontal measurements on the master casts (Table 1). A normality line test, for intraoral and master casts readings (horizontal measurements), revealed that they were not normally distributed (Fig. 4).

**Table 1:** The *t* test for horizontal measurements of the intraoral and master cast in the open and closed tray techniques

Impression technique		*N*	Mean	Std. deviation	Std. error mean	*t*	*P* value
Intraoral	Open	40	9.327	3.356	0.531	0.205	0.838
	Closed	40	9.181	2.974	0.470		
Master cast	Open	40	9.359	3.376	0.534	0.188	0.851
	Closed	40	9.225	2.970	0.470		

Significance level *p* ≤ 0.05

The Wilcoxon signed-rank test revealed that there are no statistically significant differences between the open and closed tray implant impression techniques (Table 2). The Mann-Whitney *U* test also showed no statistically significant differences for maxillary and mandibular impressions in both the open and closed tray techniques (Table 3).

**Table 2:** Open and closed tray techniques accuracy using the Wilcoxon signed-rank test

Techniques	Number of impressions	Median	Mean	SD	*P* value
Open tray	40	0.040	0.03230	0.0663	0.365
Closed tray	40	0.040	0.0437	0.918	

Significance level *p* ≤ 0.05

**Table 3:** Open and closed tray technique accuracy in the maxilla and mandible, using the Mann-Whitney *U* test

Techniques	Variables	Number of impressions	Median	Mean	SD	*P* value
Open tray	Maxilla	18	0.040	0.0833	0.076	0.107
	Mandible	22	0.030	0.0464	0.054	
Closed tray	Maxilla	18	0.040	0.0756	0.076	0.419
	Mandible	22	0.040	0.0945	0.0104	

Significance level *p* ≤ 0.05

The Mann-Whitney *U* test evaluated impression accuracy in the horizontal measurements according to the arch. There were no statistically significant differences between maxillary and mandibular arches, for the open and closed tray technique (Table 4).

**Table 4:** Impression technique accuracy in the anterior and posterior regions using the Mann-Whitney *U* test

Techniques	Variables	Number of impressions	Median	Mean	SD	*P* value
Open tray	Anterior	13	0.04	0.0569	0.0497	0.360
	Posterior	27	0.03	0.0659	0.0737	
Closed tray	Anterior	13	0.03	0.0515	0.0571	0.039*
	Posterior	27	0.04	0.1026	0.1013	

*Significance level *p* ≤ 0.05

The Mann-Whitney *U* test also showed no statistically significant difference in the horizontal measurement between the anterior and posterior regions for the open tray impression technique (Table 5). However, statistically significant differences were detected in the horizontal measurements, between the anterior and posterior regions in the closed tray impression technique (Table 4).

**Table 5:** The horizontal discrepancies according to implant position in the arch, using the Mann-Whitney *U* test

Horizontal discrepancies	Position	*N*	Open tray			Closed tray			*P* value
			Mean	Median	S.D	Mean	Median	S. D	
Maxilla	Anterior	9	0.069	0.040	0.055	0.047	0.020	0.050	0.110
	Posterior	9	0.098	0.040	0.093	0.101	0.050	0.090	0.136
	Total	18	0.084	0.040	0.075	0.075	0.035	0.070	0.584
Mandible	Anterior	4	0.030	0.035	0.022	0.063	0.079	0.030	0.999
	Posterior	18	0.050	0.030	0.059	0.102	0.109	0.045	0.118
	Total	22	0.0464	0.04	0.054	0.095	0.104	0.040	0.152

Significance level *p* ≤ 0.05

**Fig. 5:**
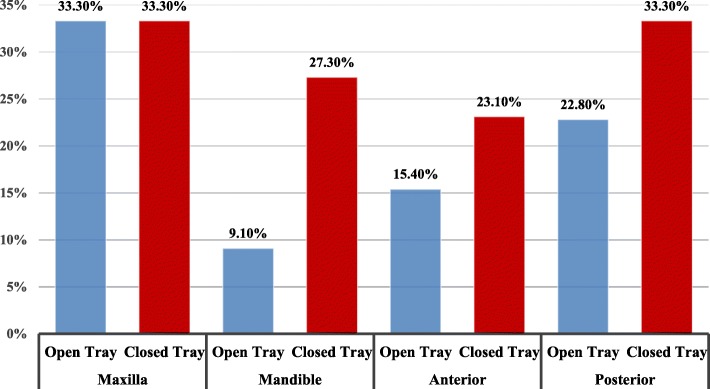
Marginal discrepancy distribution in the open and closed techniques, maxillary mandibular, and anterior and posterior regions

The marginal discrepancy evaluation and percentages by arch and region are presented in Fig. 5. The chi-square test associated marginal discrepancies between maxillary and mandibular, and anterior and posterior regions. There were no statistically significant differences in the marginal discrepancy between maxillary and mandibular arches, and anterior and posterior in both open and closed tray impression techniques (Table 6).

**Table 6:** Chi-square test of marginal discrepancies for the impression techniques, by implant position in the arch

Marginal discrepancy	Open tray	Closed tray	*P* value
Maxilla	6 (33.3%)	6 (33.3%)	0.999
Mandible	2 (9.1%)	6 (27.3%)	0.240
Anterior	2 (15.4%)	3 (23.1%)	0.50
Posterior	6 (22.2%)	9 (33.3%)	0.272

Significance level *p* ≤ 0.05

## Discussion

Impression accuracy at the implant level is believed to have a higher degree of accuracy, compared to abutment-level impressions [20]. However, studies reporting on impression accuracy in implant dentistry may vary, and there are several possible explanations for these variations. One reason is the splinting together of copings for pick-up impressions compared to the non-splinting of copings. Secondly, the edentulous spans evaluated may differ between studies; thirdly, marginal discrepancy evaluation may use magnifying visual assessment as in the current investigation, or a superimposition of digital models as in Stimmelmayr et al. [9]. One variant that might have an impact is the impression material used. In one study, the impression material had the most considerable effect size on accuracy in terms of the 3D shift, and the implant axis inclination [21].

Most of the data on implant impression accuracy is from in vitro studies, with a small number conducted in a clinical setting. The limited number of clinical studies was highlighted in a systemic review by Papaspyridakos et al., where from the 76 studies reviewed, only 4 were in a clinical setting [22]. Baig, also in a report on the accuracy of multiple implants impressions of edentulous arches, found only 3 of the 56 studies reviewed to be in a clinical setting [7]. Also, when the same author conducted a systematic review, only 1 study out of the 34 selected for the systematic review was a clinical study [23].

This prospective clinical investigation found no significant differences between open and closed tray techniques, in agreement with Gallucci et al. [24]. In our in vitro study, we also found that the open and closed tray implant impression techniques showed a similar level of accuracy [8]. In that study, all the impressions were in the posterior maxillary region, while the current study had variations of anterior, posterior, maxillary, and mandibular. However, the current study is in disagreement with Stimmelmayr et al., where they found that the splinted implants in the open tray were more accurate than that in the closed tray technique [9].

Regarding the influence on the accuracy of the implant position within the dental arch, the current study found that the implant position in the dental arch had no influence or impact on impression accuracy, similar to the report by Gallucci et al. [24]. However, and in contrast, Papaspyridakos et al. found that the position in the dental arch influenced accuracy [22]. However, the Papaspyridakos et al. study involved utilizing the open tray technique only and used splinted impression copings. Furthermore, polyether impression was the material used in their study, and accuracy evaluation was through superimposition of optical scans, and perhaps, these differences may have contributed to the variance in outcomes.

There is currently an increase in the use of digital impressions in dentistry. In a recent systematic review comparing digital and conventional impressions, out of 10 articles, 5 encouraged the use of intraoral scanners in the implant field, while two studies found that digital scanning is not reliable and could not be used in clinical practice. However, it is still early to conclude whether to use digital scanners in implant dentistry as standard procedure and further studies should clarify this issue [25].

The current study generally found no statistically significant differences in the marginal discrepancy between both impression techniques. This is contrary to the findings of Papaspyridakos et al., where statistically significant differences were found concerning marginal discrepancy between the groups studied [26].

Any stage of implant prosthesis fabrication may contribute to positional distortion or imprecision. Decreasing distortion factors in the horizontal and vertical dimensions may reduce the potential on impression misfits of the implant superstructures. Several methods may be used to evaluate the presence or absence of marginal discrepancy; in the current study, the one screw test with a verification jig was used, since it has been widely used to determine marginal discrepancies [15, 27–29].

One of the limitations of our study is the lack of matching arches and regions. This may have yielded variable data and would have perhaps influenced the outcomes of implant impression accuracy. Also, the exact position of the implant in relation to accuracy have not been considered in this study, and further studies should consider evaluating this.

A further limitation is that specialists undertook the management of the patients in this study, and it would have been interesting to evaluate the effect of clinical experience on impression accuracy, though that would have probably required a larger sample to obtain the appropriate power to assess these variables. A further limitation is the effect of implant angulations on the accuracy was not assessed in this study. Also, this study investigated impression accuracy in relationship to adjacent implants, and the results should be viewed regarding adjacent implants only, and that spaced and divergent implants would possibly yield different outcomes.

## Conclusion

Within the limitation of this study, there were no differences in the impression accuracy between the open and closed tray techniques, in partially edentulous jaws with two adjacent implants. Also, there were no differences between the two impression techniques regarding marginal discrepancy. The position of the implant, in the maxilla or mandible, had no effect on the impression accuracy of both techniques.

## Data Availability

The authors declare that they have full control on all data and materials of this study.
